# Impact of intraoperative ventilation parameters on postoperative outcomes in thoracic surgery: a multicenter registry-based analysis

**DOI:** 10.3389/fsurg.2025.1749213

**Published:** 2026-01-30

**Authors:** Timon Marvin Schnabel, Mark Schieren, Carlos Daniel Cardenas Artero, Jerome Defosse, Mark Ulrich Gerbershagen

**Affiliations:** 1 Department of Anaesthesiology, University Witten/Herdecke, Cologne-Holweide Hospital, Cologne, Germany; 2 Department of Anaesthesiology and Intensive Care Medicine, University Witten/Herdecke, Cologne-Merheim Hospital, Cologne, Germany

**Keywords:** intraoperative ventilation, lung-protective ventilation, mechanical ventilation, one-lung ventilation, pulmonary complications, thoracic surgery

## Abstract

**Objectives:**

One-lung ventilation (OLV) is a standard technique during thoracic surgery, yet its impact on postoperative complications and ventilator settings remains under investigation. The objective of this study was to evaluate the impact of intraoperative ventilation parameters on postoperative outcomes in patients undergoing thoracic surgery with OLV.

**Design and setting:**

A retrospective multicenter cohort analysis was conducted using data from the German Thoracic Registry.

**Participants:**

The study encompassed 2,922 patients treated between 2017 and 2021 across eight German centers.

**Interventions:**

Intraoperative variables analyzed included driving pressure (DP), positive end-expiratory pressure (PEEP), maximum airway pressure (pMax), tidal volume (TV) per predicted body weight (PBW), and ventilation mode. The primary outcomes of interest were postoperative complications, respiratory complications, and in-hospital mortality.

**Measurements and main results:**

Postoperative complications occurred in 28.7% of cases. Elevated DP (>20 mbar), pMax (>25 mbar), and PEEP (>8 mbar) were significantly associated with increased complication and mortality rates. Patients receiving a TV > 5 mL/kg PBW also showed higher complication rates (*p* = .003). Respiratory complications occurred in 15.7% of patients and were strongly associated with higher DP, pMax, and OLV duration. Multivariate logistic regression identified OLV > 60 min and pMax >25 mbar as independent predictors of respiratory complications and overall complications.

**Conclusion:**

Intraoperative ventilation parameters, particularly elevated DP, pMax and PEEP, have been demonstrated to be associated with an increased risk of complications and mortality in patients undergoing thoracic surgery with OLV. These findings lend support to the hypothesis that lung-protective ventilation strategies may improve perioperative outcomes.

## 1 Introduction

Lung cancer is responsible for a significant proportion of cancer-related deaths worldwide (1). Annually, approximately 57,000 individuals are diagnosed with lung cancer in Germany, and five-year survival rates remain low at 19% for males and 25% for females in 2022 (2). In light of the observed demographic shifts and the mounting prevalence of environmental and occupational risk factors, there is a compelling argument to be made that the global burden of lung cancer is poised to escalate further in the ensuing decades (3–7).

Despite the advances that have been made in systemic therapies, surgical resection remains the mainstay of curative treatment for early-stage lung cancer (8–10). However, thoracic surgery is frequently associated with postoperative pulmonary complications, which include, but are not limited to, pneumonia, atelectasis, respiratory insufficiency and acute respiratory distress syndrome (ARDS) (11–13). These complications are of particular concern following procedures involving one-lung ventilation (OLV) (14, 15).

The most frequently employed method for facilitating OLV is by means of the utilization of double-lumen tubes or bronchial blockers, with the objective being to enable lung isolation (16). While this technique provides optimal surgical conditions, it imposes increased mechanical and inflammatory stress on the ventilated lung, raising concerns about ventilator-associated lung injury and hypoxemia (17–19).

Over the past two decades, lung-protective ventilation (LPV) has become a cornerstone in the management of mechanically ventilated patients, particularly in intensive care settings (20). However, its intraoperative implementation remains inconsistent across centers, particularly in the context of one-lung ventilation in thoracic surgery (21).

Mechanical ventilation parameters, such as tidal volume (TV) per kg predicted body weight (PBW), positive end-expiratory pressure (PEEP), driving pressure (DP) and maximum airway pressure (pMax) have now been established as critical determinants of postoperative outcomes. Nevertheless, the optimal settings for OLV remain the subject of ongoing debate (14, 22–26).

This study aims to investigate the relationship between intraoperative ventilation parameters and postoperative complications in patients undergoing thoracic surgery with OLV, based on data from the German Thoracic Register. By identifying intraoperative thresholds associated with elevated risk, the study seeks to support the development of standardized ventilation protocols that may reduce postoperative pulmonary complications, shorten hospital stays, and improve survival.

## 2 Materials and methods

### 2.1 Study design

The present study was designed as a retrospective, multicenter cohort analysis conducted at eight German thoracic surgical centers. The analysis was based on data recorded in the German Thoracic Registry (GTR) between January 2017 and December 2021. The registry provides a comprehensive, standardized dataset covering the entire perioperative course of thoracic surgical patients, encompassing preoperative, intraoperative, and postoperative information. All participating centers perform ≥50 thoracic surgical procedures annually.

The present investigation sought to examine the association between intraoperative ventilation parameters during one-lung ventilation (OLV) and the incidence of postoperative complications, respiratory complications, and in-hospital mortality.

### 2.2 Patient selection

**Figure 1:**
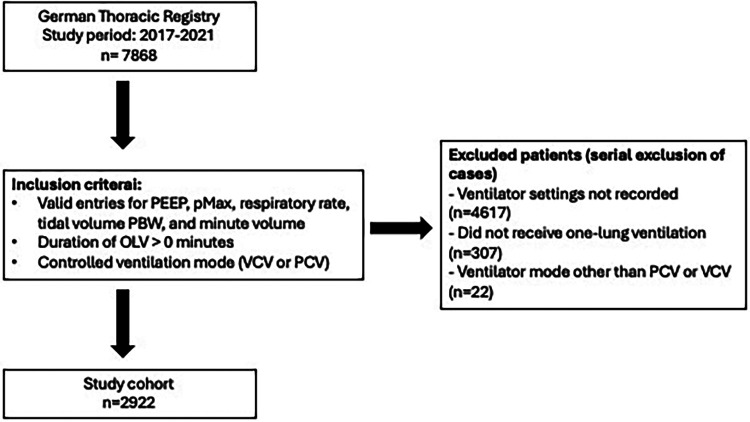
Flowchart of inclusion and exclusion criteria. PEEP, positive end-expiratory pressure; pMax, maximum airway pressure; PBW, predicted body weight; OLV, one-lung ventilation; VCV, volume-controlled ventilation; PCV, pressure-controlled ventilation.

Inclusion criteria comprised all adult patients who underwent thoracic surgery with intraoperative OLV in a controlled ventilator mode and complete documentation of the ventilator settings. Inclusion and exclusion criteria are shown in Figure 1.

### 2.3 Ventilation and surgical parameters

The following intraoperative ventilation parameters were extracted from the protocol:

DP: Calculated as Pmax—PEEP
PEEP
pMax
TV/PBW
Ventilation Mode: PCV or VCV

The surgical variables that were considered in this study included the type and extent of resection (e.g., wedge resection, lobectomy, pneumonectomy), surgical approach [video-assisted thoracoscopic surgery (VATS) vs. thoracotomy], duration of surgery, and duration of OLV. These variables were considered as potential covariates in the multivariable analyses. Airway management was documented (double-lumen tube, bronchial blocker).

### 2.4 Outcome parameter

The primary outcomes were defined as the occurrence of postoperative complications, which were characterized as any deviation from the expected clinical course necessitating medical intervention.

Respiratory complications were defined as a composite endpoint including pneumonia, respiratory insufficiency, ARDS, bronchial stump insufficiency, bronchopleural fistula >7 days, re-intubation, non-invasive ventilation (NIV), extracorporeal membrane oxygenation (ECMO) or unplanned Intensive Care Unit (ICU) admission.

The secondary outcome was in-hospital mortality, defined as all-cause death during the index hospitalization.

### 2.5 Data collection

The data were prospectively recorded in the GTR using a standardized, pseudonymized electronic data sheet and subsequently extracted for statistical analysis. All surgical procedures were carried out in accordance with institutional guidelines for quality assurance in perioperative medicine.

The preoperative variables encompassed age, sex, body mass index (BMI), American Society of Anesthesiologists (ASA) status, smoking history, comorbidities (including diabetes, renal insufficiency, cardiovascular disease, peripheral arterial disease, cerebrovascular disease, obstructive sleep apnea syndrome, and chronic pain syndrome), preoperative pulmonary function parameters (FEV1) and laboratory parameters. Intraoperative data included ventilation parameters, surgical duration, and airway management. Postoperative outcomes were meticulously documented until patients were discharged from hospital or died during hospitalization. Information pertaining to preoperative oncologic therapy (chemotherapy and/or radiotherapy) and previous lung surgery was also collated and is summarized in Table 1.

**Table 1:** Demographic data.

Category	Variable	*n* (%)
Total patients	–	2,922
Gender	Male	1,750 (59.9%)
	Female	1,172 (40.1%)
Smoking status	Never smoked	941 (32.2%)
	Current smokers	865 (29.6%)
	Former smokers (quit >3 months)	1,116 (38.2%)
Preoperative respiratory infection	≤4 weeks before surgery	193 (6.6%)
Comorbidities	Diabetes mellitus	383 (13.1%)
	Renal insufficiency	278 (9.5%)
	Coronary artery disease (CAD)	430 (14.7%)
	Peripheral arterial disease (PAD)	167 (5.7%)
	Stroke/Transient ischemic attack (TIA)	152 (5.2%)
	Obstructive sleep apnea syndrome (OSAS)	108 (3.7%)
	Chronic pain syndrome	140 (4.8%)
Preoperative oncologic therapy	No preoperative therapy	2,267 (77.6%)
	Chemotherapy	386 (13.2%)
	Radiotherapy	152 (5.2%)
	Previous lung surgery	421 (14.4%)
ASA—Classification	ASA 1	37 (1.3%)
	ASA 2	626 (21.4%)
	ASA 3	2,008 (68.7%)
	ASA 4	245 (8.4%)
	ASA 5	6 (0.2%)
BMI	Mean	26.69 (SD 5.08)
FEV1	Mean	80.95 (SD 21.04)

### 2.6 Statistical analysis

A comprehensive set of descriptive statistics was derived for all baseline and procedural variables. Categorical variables were expressed as absolute and relative frequencies. Chi-squared (*χ*^2^) tests were used to assess the associations between stratified ventilation parameters and categorical outcomes (complications, respiratory complications, mortality).

For continuous variables, parametric and non-parametric tests (*t*-test and Mann–Whitney *U*-test) were applied as appropriate. An exploratory data analysis was conducted to identify thresholds for increased risk associated with ventilation parameters, considering both existing literature and clinical relevance.

Multivariable logistic regression models were applied in order to identify independent predictors of postoperative complications and mortality, with adjustments made for age, sex, ASA status, surgical type (anatomical vs. non-anatomical resection), and ventilation mode.

It is important to note that all statistical tests were two-tailed, with a significance level of *p* < .05. Statistical analyses were performed using SPSS Version 25.0.

### 2.7 Ethical considerations

The study was approved by the institutional review board of the University of Witten/Herdecke (approval no. 64-2014). The data were subsequently pseudonymized in accordance with the General Data Protection Regulation guidelines. Written informed consent was obtained from all patients before enrollment in the GTR.

## 3 Results

### 3.1 Total demographic data

The study was predominantly composed of male patients. A considerable proportion of the subjects were former smokers, followed by current smokers and those who had never smoked. A small percentage of the sample had experienced a preoperative respiratory infection. Mean preoperative FEV1 was 80.95 (SD 21.04), indicating that baseline pulmonary function was moderately preserved in most patients.

The prevalence of comorbidities such as diabetes, renal insufficiency, coronary artery disease, peripheral arterial disease, stroke or transient ischemic attack, obstructive sleep apnea syndrome, and chronic pain syndrome was also observed.

The majority of patients had not undergone preoperative oncological therapy (77.6%), whereas 13.2% had received chemotherapy and 5.2% radiotherapy. Documentation pertaining to previous thoracic surgery was found in 14.4% of cases. The complete set of data is displayed in Table 1.

### 3.2 Total complications

Out of 2,922 patients, 28.7% (*n* = 839) experienced complications. Several ventilation parameters were associated with a significantly higher complication rate.

An increase in DP above 15 mbar was found to be associated with a significant rise in complication rates from 26.5% to 32.4% (*p* < .001). In a similar vein, when DP exceeded 20 mbar, complication rates increased from 27.9% to 36.2% (*p* = .004).

With regard to pMax, a threshold of 20 mbar was found to be associated with an increased complication rate from 26.2% to 31.4% (*p* = .002). When pMax exceeded 25 mbar, the complication rate exhibited a marked increase from 27.1% to 37.9% (*p* < .001). Furthermore, pMax values greater than 30 mbar were associated with an elevated complication rate from 28.3% to 44.2% (*p* = .002).

A PEEP level in excess of 8 mbar was found to be associated with an increased complication rate from 28.0% to 36.1% (*p* = .008). In a similar manner, a PEEP threshold of 7 mbar corresponded to an increase in complication rates from 27.6% to 33.8% (*p* = .004).

Finally, a TV greater than 5 mL/kg PBW was found to be significantly associated with an increased complication rate from 23.9% to 30.0% (*p* = .003).

**Figure 2:**
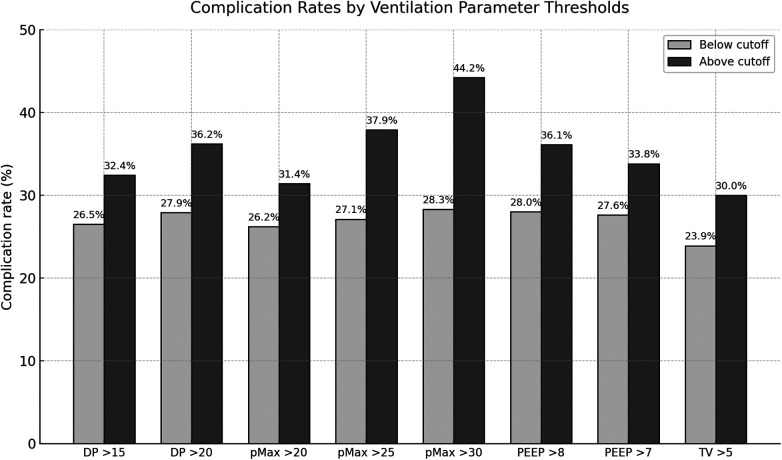
Postoperative complication rates in patients below and above predefined ventilation parameter cutoffs during one-lung ventilation. DP, driving pressure; pMax, maximum airway pressure; PEEP, positive end-expiratory pressure; TV, tidal volume.

Complete results for total complications are presented in Figure 2 and Table 2.

**Table 2:** Results of total complications in total cohort.

Parameter	Comparison value (%)	Complication rate (%)	*Χ*^2^ (df = 1)	*p*-value
DP > 15 mbar	26.5	32.4	11.82	<.001
DP > 20 mbar	27.9	36.2	8.45	.004
pMax > 20 mbar	26.2	31.4	9.53	.002
pMax > mbar	27.1	37.9	21.24	<.001
pMax > 30 mbar	28.3	44.2	9.21	.002
PEEP > 8 mbar	28.0	36.1	7.03	.008
PEEP > 7 mbar	27.6	33.8	8.29	.004
TV > 5 mL/kg PBW	23.9	30.0	8.58	.003

DP, driving pressure; pMax, maximum airway pressure; PEEP, positive end-expiratory pressure; TV, tidal volume; df, degrees of freedom.

A binary logistic regression was performed on 2,922 patients to identify predictors of postoperative complications following thoracic surgery with OLV. The model was statistically significant (*χ*^2^(6) = 144.14, *p* < .001), explained 6.9% of the variance (Nagelkerke *R*^2^), and correctly classified 71.5% of cases. Significant predictors included anatomical resection (*p* = .006), OLV duration >60 min (*p* < .001), peak pressure >25 mbar (*p* = .002), and TV >5 mL/kg PBW (*p* < .001). Non-significant predictors were PEEP > 7 mbar (*p* = .065) and DP > 15 mbar (*p* = .303).

### 3.3 Respiratory complications

A total of 15.7% (*n* = 460) experienced respiratory complications.

Elevated DP has been demonstrated to be a significant predictor of an increased complication rate. Specifically, when DP exceeded 15 mbar, the complication rate increased from 14.1% to 18.4% (*p* = .002). In a similar manner, a threshold of 20 mbar was associated with an increase from 15.2% to 21.5% (*p* = .020), and DP values greater than 25 mbar were linked to an increase from 15.6% to 27.9% (*p* = .027).

Regarding pMax, significant differences were also observed. A rise in pressure greater than 20 mbar was found to result in a higher complication rate, from 13.8% to 17.8% (*p* = .003). Similarly, pMax values exceeding 25 mbar were found to be associated with a marked increase in the complication rate, from 14.5% to 22.8% (*p* < .001). Furthermore, when pMax exceeded 30 mbar, complication rates increased from 15.5% to 24.7% (*p* = .029).

Furthermore, PEEP demonstrated a noteworthy correlation with complication rates. A PEEP level in excess of 8 mbar was found to be associated with an increase from 15.3% to 20.5% (*p* = .033). In a similar vein, when PEEP exceeded 7 mbar, the complication rate increased from 15.0% to 19.0% (*p* = .021). The complete results have been collated and are displayed in Table 3.

**Table 3:** Results of respiratory complications in total cohort.

Parameter	Comparison value (%)	Complication rate (%)	*χ*^2^(df = 1)	*p*-value
DP > 15 mbar	14.1	18.4	9.34	.002
DP > 20 mbar	15.2	21.5	7.85	.020
DP > 25 mbar	15.6	27.9	4.87	.027
pMax > 20 mbar	13.8	17.8	8.90	.003
pMax > 25 mbar	14.5	22.8	19.52	<.001
pMax > 30 mbar	15.5	24.7	4.76	.029
PEEP > 8 mbar	15.3	20.5	4.53	.033
PEEP > mbar	15.0	19.0	5.34	.021

DP, driving pressure; pMax, maximum airway pressure; PEEP, positive end-expiratory pressure; df, degrees of freedom.

Increased duration of OLV and surgery also showed a strong correlation with respiratory complications. Patients with OLV durations >180 min had a 33.2% complication rate vs. 6.6% at ≤30 min (*χ*^2^(6) = 156.6, *p* < .001). Similar patterns were observed for longer surgery durations (*χ*^2^(6) = 181.0, *p* < .001).

A binary logistic regression was conducted to identify predictors of respiratory complications in 2,922 thoracic surgery patients. The outcome was a composite of major postoperative respiratory complications. The model was statistically significant (*χ*^2^(6) = 122.53, *p* < .001), explained 7.1% of the variance (Nagelkerke *R*^2^), and correctly classified 84.3% of cases. Significant predictors included anatomical resection (*p* < .001), OLV duration >60 min (*p* < .001), peak pressure >25 mbar (*p* = .001), and TV >5 mL/kg PBW (*p* = .028). Non-significant predictors were PEEP > 7 mbar (*p* = .117) and DP > 15 mbar (*p* = .468).

### 3.4 Overall mortality

Among the total cohort, 97.9% of patients were discharged alive, while 2.1% (*n* = 62) died during hospitalization. A number of ventilation parameters were found to be significantly associated with elevated mortality rates.

An increase in DP above 20 mbar was found to be associated with a significant rise in mortality from 1.8% to 5.4% (*p* < .001).

In a similar vein, elevated pMax exhibited a substantial correlation with mortality. When pMax exceeded 20 mbar, the mortality rate increased from 1.5% to 2.8% (*p* = .019). A pMax greater than 25 mbar was associated with an elevated mortality rate from 1.6% to 5.0% (*p* < .001). Furthermore, when pMax exceeded 30 mbar, there was a marked increase in mortality from 1.8% to 13.0% (*p* < .001).

Furthermore, PEEP has been demonstrated to have a significant impact on mortality rates. The presence of PEEP values in excess of 8 mbar has been demonstrated to be associated with an increase in mortality, from 1.9% to 4.9% (*p* = .002). In a similar manner, a PEEP threshold of 7 mbar was associated with an increase in mortality from 1.7% to 3.9% (*p* = .002). The complete results of the mortality study are displayed in Table 4.

**Table 4:** Results of mortality in total cohort.

Parameter	Comparison value (%)	Complication rate (%)	χ^2^(df = 1)	*p*-value
DP > 20 mbar	1.8	5.4	15.73	.001
pMax > 20 mbar	1.5	2.8	5.50	.019
pMax > 25 mbar	1.6	5.0	20.88	.001
pMax > 30 mbar	1.8	13.0	44.95	.001
PEEP > 8 mbar	1.9	4.9	10.02	<.002
PEEP > 7 mbar	1.7	3.9	9.84	.002

DP, driving pressure; pMax, maximum airway pressure; PEEP, positive end-expiratory pressure; df, degrees of freedom.

Longer durations of both OLV and surgery were also significantly associated with increased mortality (all *p* < .001).

**Figure 3:**
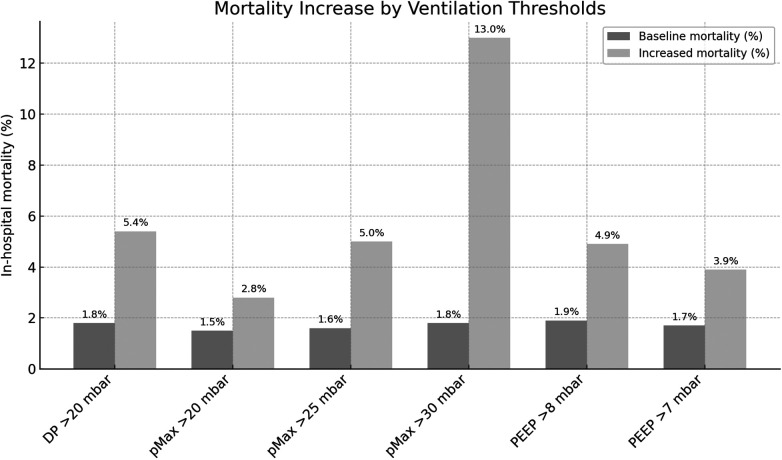
In-hospital mortality rates stratified by ventilation parameters. DP, driving pressure; pMax, maximum airway pressure; PEEP, positive end-expiratory pressure; TV, tidal volume.

Results for total in—hospital Mortality are shown in Figure 3.

**Figure 4:**
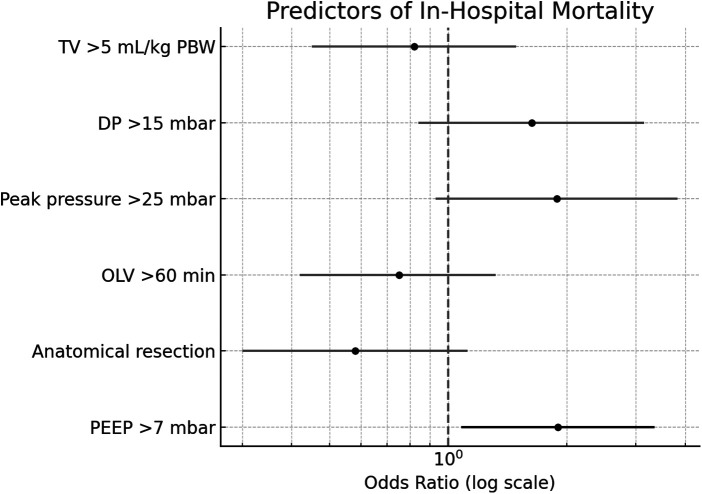
Forest plot displaying odds ratios and 95% confidence intervals for predictors of in-hospital mortality. Only PEEP >7 mbar was a statistically significant predictor (black error bar). Gray bars indicate non-significant predictors. TV, tidal volume; DP, driving pressure; OLV, one lung ventilation; PEEP, positive end-expiratory pressure.

A binary logistic regression was conducted to identify predictors of in-hospital mortality among 2,922 thoracic surgery patients. The model was statistically significant (*χ*^2^(6) = 29.24, *p* < .001), explained 5.4% of the variance (Nagelkerke *R*^2^), and correctly classified 97.9% of cases. The only significant predictor was PEEP > 7 mbar (*p* = .026). Non-significant predictors included anatomical resection (*p* = .103), OLV duration >60 min (*p* = .315), peak pressure >25 mbar (*p* = .078), DP >15 mbar (*p* = .146), and TV > 5 mL/kg PBW (*p* = .512). Results of the Regression analysis are shown in Figure 4.

## 4 Discussion

The present study demonstrated an association between elevated parameters of intraoperative ventilation, particularly DP, pMax, PEEP and TV > 5 mL/kg PBW and increased rates of postoperative complications, respiratory complications and in-hospital mortality in patients receiving thoracic surgery with OLV. The total cohort demonstrated a consistent relationship between elevated ventilation parameters and complication rates. Prolonged OLV (>60 min) and elevated pMax (>25 mbar) were identified as significant predictors of complications and respiratory failure. Notwithstanding multivariable analysis and adjustment for surgical type and ASA classification, these findings remained consistent. However, the duration of OLV is predominantly influenced by the surgical procedure itself, the extent of resection, and the invasiveness of the operation. Consequently, it should be primarily interpreted as a surrogate marker of case complexity rather than a directly modifiable ventilatory target.

These observations align with the established concept of intraoperative lung-protective ventilation during OLV in thoracic surgery, thereby largely corroborating current practices in numerous high-volume thoracic centers. The present analysis has added value in confirming these associations in a large, prospectively collected multicenter registry cohort, thereby strengthening the external validity and generalizability of existing evidence.

Elevated pMax (>25–30 mbar), DP (>20 mbar), and TV > 5 mL/kg PBW have been shown to be associated with higher complication rates. This finding aligns with the principles of LPV as established in intensive care and perioperative research (17, 21, 27, 28).

It is evident that previous research in this field placed emphasis on the utilization of reduced TV and the optimization of PEEP and DP (3, 14, 22–24). Nevertheless, a potential limitation of these studies was the omission of standardized multicenter data collection shown in this study (29).

The in-hospital mortality rate of 2.1% is commensurate with the expected range for thoracic surgery populations and is analogous to rates documented in previous studies (1.1%–5%) (30–32). However, the striking increase in mortality with high pMax values—reaching 13.0% at pMax >30 mbar in the overall cohort suggests a potential dose-dependent relationship. Given the retrospective nature of the data, this association should be interpreted with caution, as causality cannot be established. A number of studies have previously observed an association between OLV intensity and respiratory complications (14, 15), the association between increased airway pressure and mortality in OLV remains a subject of discussion (28, 33, 34). It is noteworthy that in this study, PEEP > 7 mbar was the sole parameter that maintained statistical significance in the multivariable mortality model (OR = 1.90, *p* = .026), indicating a potential adverse hemodynamic or overdistension effect at higher levels during OLV. While not all parameters were predictive of mortality, their association with complications was consistent, underscoring their clinical relevance even in the absence of direct mortality impact.

The present study possesses several notable methodological strengths. Firstly, the large-scale, multicenter dataset encompassing 2,922 patients enhances both statistical power and the generalizability of the findings. It is important to note that the data were derived from the GTR, a national registry, that has been standardized to ensure consistent and high-quality data collection across participating centers. This prospective standardized approach of data collection serves to minimize the impact of bias in documentation and facilitates robust cross-center comparisons. Furthermore, the employment of stratified analyses and multivariable regression models enabled the adjustment of key confounders, including surgical procedure type, ASA status classification, and ventilation mode.

The present study is subject to several limitations. Although the study was designed as a retrospective cohort analysis, it is based on prospectively and systematically collected data from the GTR, enhancing the internal validity and reducing information bias. The ventilation parameters exhibited significant variability across centers, potentially reflecting individual clinician preferences or patient-specific considerations. Despite the fact that only patients with complete documentation of key ventilation variables were included in the study, the potential for missing data bias and selection bias remains. Furthermore, critical intraoperative parameters, such as plateau pressure and static lung compliance were unavailable, thereby limiting the capacity to accurately characterize mechanical lung stress. The study also lacked data on postoperative ventilator settings and ICU care practices, which may influence patient outcomes and complicate interpretation of intraoperative effects in isolation. Furthermore, the preoperative pulmonary function in the present analysis was primarily summarized by FEV1. The multivariable models did not incorporate other spirometric indices, such as forced vital capacity and diffusing capacity for carbon monoxide. Consequently, it was not possible to formally assess whether impaired baseline respiratory function modified the relationship between intraoperative ventilation intensity and postoperative respiratory complications. Of particular note, chronic obstructive pulmonary disease (COPD), a prevalent comorbidity in thoracic surgical patients and a salient factor in ventilator management, was not systematically coded in the registry dataset. Consequently, it could not be incorporated into the present models, an omission that may have led to residual confounding. Additionally, the present study did not incorporate stratified analyses according to specific underlying pulmonary diseases, preoperative oncologic treatment, or prior lung surgery. Nor did it distinguish between malignant and non-malignant etiologies or between elective and urgent procedures, as these variables were not available in a standardized form across centers. These factors may influence susceptibility to ventilator-induced lung injury and postoperative respiratory failure, and future studies should specifically address their interaction with intraoperative ventilation strategies. Moreover, the data pertaining to inflammatory laboratory values were absent, thereby rendering it impossible to identify any potential correlations between intraoperative ventilation parameters and systemic inflammatory responses. This also precluded the validation of pathophysiological mechanisms such as biotrauma or cytokine-mediated lung injury. The cohort comprised a broad spectrum of thoracic procedures, ranging from wedge resections to pneumonectomies and including both VATS and open approaches. Despite the fact that surgical type (anatomical vs. non-anatomical resection) was included as a covariate in the multivariable models, detailed procedure-specific subgroup analyses were not presented. Analyses of this kind have the potential to offer significant clinical insights, given the notable disparities in respiratory and anesthetic stress observed between pneumonectomy and wedge resection procedures. It is therefore recommended that this aspect be given due consideration in forthcoming investigations.

Notwithstanding the limitations previously mentioned, the study identifies specific intraoperative ventilation thresholds, such as pMax > 25 mbar and tidal volumes >5 mL/kg predicted body weight, that are associated with an increased risk of postoperative complications and mortality. The findings of this study provide clear, clinically actionable targets and reinforce the importance of standardized intraoperative LPV protocols, particularly in OLV. The data support the use of individualized ventilation strategies, including driving pressure-guided approaches, to mitigate risk in thoracic anesthesia. Furthermore, the findings indicate that reducing ventilation duration and incorporating recruitment maneuvers or alternative modalities may prove particularly advantageous in protracted procedures.

It is recommended that future studies concentrate on the execution of prospective, randomized controlled trials. The purpose of such studies would be to validate the observed associations and establish causal links between intraoperative ventilation strategies and postoperative outcomes. In order to elucidate the pathophysiological pathways underlying ventilator-induced lung injury, mechanistic investigations incorporating dynamic lung compliance measurements, inflammatory biomarkers, and real-time imaging techniques (e.g., lung ultrasound or CT-based aeration analysis) are required. Furthermore, the development of real-time optimization algorithms or artificial intelligence–assisted decision support systems may enhance intraoperative ventilation management. Longitudinal studies assessing the impact of protective intraoperative ventilation on long-term outcomes, including pulmonary function and quality of life, are also recommended.

In summary, this multicenter registry analysis reinforces the pivotal role of LPV during OLV and identifies specific intraoperative parameters—namely pMax, DP, and TV—that independently predict adverse outcomes. These data strengthen the argument for recent calls for consensus guidelines on intraoperative LPV in thoracic surgery and may serve as a benchmark for perioperative quality assurance. While further prospective validation remains essential, current evidence suggests that limiting peak pressures and TV and avoiding unnecessarily prolonged OLV where surgically feasible, could meaningfully reduce postoperative morbidity and mortality in this high-risk patient population.

## 5 Conclusion

This multicenter analysis identifies elevated intraoperative ventilation parameters—particularly pMax > 25 mbar and TV > 5 mL/kg PBW—as independent predictors of postoperative complications, respiratory failure, and mortality in thoracic surgery with OLV. These findings provide a robust rationale for the implementation of standardized lung-protective ventilation strategies that are tailored to intraoperative risk thresholds.

## Data Availability

The data analyzed in this study is subject to the following licenses/restrictions: The dataset consists of pseudonymized patient-level data from the German Thoracic Registry and is subject to institutional data use agreements, registry governance, and European data protection regulations. For these reasons, the raw data cannot be made publicly available or deposited in an open repository; access for external researchers would require prior approval by the registry steering committee and the responsible ethics committee and can only be granted in aggregated or further de-identified form. Requests to access these datasets should be directed to timonschnabel1@gmx.de.

## References

[1] World Health Organisation [WHO]. Lung cancer (2023).

[2] Robert Koch Institut, Zentrum für Krebsregisterdaten. Lung cancer (2024). Available online at: https://www.krebsdaten.de/Krebs/EN/Content/Cancer_sites/Lung_cancer/lung_cancer_node.html (Accessed April 24, 2024)

[3] Zhang Y Vaccarella S Morgan E Li M Etxeberria J Chokunonga E Global variations in lung cancer incidence by histological subtype in 2020: a population-based study. Lancet Oncol. (2023) 24(11):1206–18. 10.1016/S1470-2045(23)00444-8 37837979

[4] Wéber A Morgan E Vignat J Laversanne M Pizzato M Rumgay H Lung cancer mortality in the wake of the changing smoking epidemic: a descriptive study of the global burden in 2020 and 2040. BMJ Open. (2023) 13(5):e065303. 10.1136/bmjopen-2022-065303 PMC10174019 37164477

[5] Wan W Peters S Portengen L Olsson A Schüz J Ahrens W Occupational benzene exposure and lung cancer risk: a pooled analysis of 14 case-control studies. Am J Respir Crit Care Med. (2024) 209(2):185–96. 10.1164/rccm.202306-0942OC 37812782 PMC10806413

[6] Wang Z Cai XJ Shi L Li FY Lin NM. Risk factors of postoperative nosocomial pneumonia in stage I-IIIa lung cancer patients. Asian Pac J Cancer Prev. (2014) 15(7):3071–4. 10.7314/APJCP.2014.15.7.3071 24815449

[7] Kratzer TB Bandi P Freedman ND Smith RA Travis WD Jemal A Lung cancer statistics, 2023. Cancer. (2024) 130(8):1330–48. 10.1002/cncr.35128 38279776

[8] Cannone G Comacchio GM Pasello G Faccioli E Schiavon M Dell’Amore A Precision surgery in NSCLC. Cancers (Basel). (2023) 15(5):1571. 10.3390/cancers15051571 36900362 PMC10000462

[9] Raman V Jawitz OK Yang CFJ Voigt SL Wang H Amico D Outcomes of surgery versus chemoradiotherapy in patients with clinical or pathologic stage N3 non–small cell lung cancer. J Thorac Cardiovasc Surg. (2019) 158(6):1680–1692.e2. 10.1016/j.jtcvs.2019.08.033 31606169 PMC7311926

[10] Deutsche Krebsgesellschaft e.V. (DKG). Prävention, Diagnostik, Therapie und Nachsorge des Lungenkarzinoms Version 4.0. Berlin: Leitlinienprogramm Onkologie / Deutsche Krebsgesellschaft e. V (2025) pp. 238–302.

[11] Simonsen DF Søgaard M Bozi I Horsburgh CR Thomsen RW. Risk factors for postoperative pneumonia after lung cancer surgery and impact of pneumonia on survival. Respir Med. (2015) 109(10):1340–6. 10.1016/j.rmed.2015.07.008 26209227

[12] Rotman JA Plodkowski AJ Hayes SA de Groot PM Shepard JAO Munden RF Postoperative complications after thoracic surgery for lung cancer. Clin Imaging. (2015) 39(5):735–49. 10.1016/j.clinimag.2015.05.013 26117564

[13] Roungeris L Devadze G Talliou C Griva P. Prediction of postoperative complications after Major lung resection: a literature review. Anesth Res. (2024) 1(2):146–56. 10.3390/anesthres1020014

[14] Piccioni F Langiano N Bignami E Guarnieri M Proto P D’Andrea R One-Lung ventilation and postoperative pulmonary complications after Major lung resection surgery. A multicenter randomized controlled trial. J Cardiothorac Vasc Anesth. (2023) 37(12):2561–71. 10.1053/j.jvca.2023.04.029 37730455 PMC10133024

[15] Suleiman A Azizi BA Munoz-Acuna R Ahrens E Tartler TM Wachtendorf LJ Intensity of one-lung ventilation and postoperative respiratory failure: a hospital registry study. Anaesth Crit Care Pain Med. (2023) 42(5):101250. 10.1016/j.accpm.2023.101250 37236317

[16] Ashok V Francis J. A practical approach to adult one-lung ventilation. BJA Educ. (2018) 18(3):69–74. 10.1016/j.bjae.2017.11.007 33456813 PMC7808029

[17] Lohser J Slinger P. Lung injury after one-lung ventilation: a review of the pathophysiologic mechanisms affecting the ventilated and the collapsed lung. Anesth Analg. (2015) 121(2):302–18. 10.1213/ANE.0000000000000808 26197368

[18] Shum S Huang A Slinger P. Hypoxaemia during one lung ventilation. BJA Educ. (2023) 23(9):328–36. 10.1016/j.bjae.2023.05.006 37600211 PMC10435364

[19] Ruijiao Z Tianyuan L Shiyin W Sihui M Shumei D Lei X One lung ventilation during thoracoscopic lobectomy alters lung microbiome diversity and composition. Sci Rep. (2025) 15(1):4937. 10.1038/s41598-025-89233-4 39929955 PMC11811219

[20] Hoshino T Yoshida T. Future directions of lung-protective ventilation strategies in acute respiratory distress syndrome. Acute Med Surg. (2024) 11(1):e918. 10.1002/ams2.918 38174326 PMC10761614

[21] Hu X Du B. Lung-protective ventilation during one-lung ventilation: known knowns, and known unknowns. J Thorac Dis. (2019) 11(Suppl 3):237–40. 10.21037/jtd.2019.01.45 PMC6424720 30997186

[22] Kuo CY Liu YT Chen TS Lam CF Wu MC. A nationwide survey of intraoperative management for one-lung ventilation in Taiwan: time to accountable for diversity in protective lung ventilation. BMC Anesthesiol. (2020) 20(1):236. 10.1186/s12871-020-01157-w 32938385 PMC7493315

[23] Marret E Cinotti R Berard L Piriou V Jobard J Barrucand B Protective ventilation during anaesthesia reduces major postoperative complications after lung cancer surgery: a double-blind randomised controlled trial. Eur J Anaesthesiol EJA. (2018) 35(10):727–35. 10.1097/EJA.0000000000000804 29561278

[24] Gu WJ Zhao FZ Piccioni F Shi R Si X Chen S Individualized PEEP titration by lung compliance during one-lung ventilation: a meta-analysis. Crit Care. (2025) 29(1):27. 10.1186/s13054-024-05237-y 39825438 PMC11740579

[25] Szegedi LL. Pathophysiology of one-lung ventilation. Anesthesiol Clin N Am. (2001) 19(3):435–53. 10.1016/S0889-8537(05)70242-X 11571901

[26] Feng G Jia Y Zhao G Meng F Wang T. Risk factors for postoperative pulmonary complications in elderly patients undergoing video-assisted thoracoscopic surgery lobectomy under general anesthesia: a retrospective study. BMC Surg. (2024) 24(1):153. 10.1186/s12893-024-02444-w 38745149 PMC11091990

[27] Montes FR Pardo DF Charrís H Tellez LJ Garzón JC Osorio C. Comparison of two protective lung ventilatory regimes on oxygenation during one-lung ventilation: a randomized controlled trial. J Cardiothorac Surg. (2010) 5(1):99. 10.1186/1749-8090-5-99 21044330 PMC2987929

[28] Blank RS Colquhoun DA Durieux ME Kozower BD McMurry TL Bender SP Management of one-lung ventilation: impact of tidal volume on complications after thoracic surgery. Anesthesiology. (2016) 124(6):1286–95. 10.1097/ALN.0000000000001100 27011307

[29] Colquhoun DA Naik BI Durieux ME Shanks AM Kheterpal S Bender SP Management of 1-lung ventilation—variation and trends in clinical practice: a report from the multicenter perioperative outcomes group. Anesth Analg. (2018) 126(2):495–502. 10.1213/ANE.0000000000002642 29210790 PMC5836497

[30] Neto AS Hemmes SN Barbas CS Beiderlinden M Fernandez-Bustamante A Futier E Incidence of mortality and morbidity related to postoperative lung injury in patients who have undergone abdominal or thoracic surgery: a systematic review and meta-analysis. Lancet Respir Med. (2014) 2(12):1007–15. 10.1016/S2213-2600(14)70228-0 25466352

[31] Cheng YD Gao Y Zhang H Duan CJ Zhang CF. Effects of OLV preconditioning and postconditioning on lung injury in thoracotomy. Asian J Surg. (2014) 37(2):80–5. 10.1016/j.asjsur.2013.09.003 24315399

[32] Ferguson MK Watson S Johnson E Vigneswaran WT. Predicted postoperative lung function is associated with all-cause long-term mortality after major lung resection for cancer†. Eur J Cardiothorac Surg. (2014) 45(4):660–4. 10.1093/ejcts/ezt462 24052607 PMC4416119

[33] Liu HM Zhang GW Yu H Li XF Yu H. Association between mechanical power during one-lung ventilation and pulmonary complications after thoracoscopic lung resection surgery: a prospective observational study. BMC Anesthesiol. (2024) 24(1):176. 10.1186/s12871-024-02562-1 38760677 PMC11100229

[34] Zhu YQ Fang F Ling XM Huang J Cang J. Pressure-controlled versus volume-controlled ventilation during one-lung ventilation for video-assisted thoracoscopic lobectomy. J Thorac Dis. (2017) 9(5):1303. 10.21037/jtd.2017.04.36 28616282 PMC5465153

